# Detection of gene orthology from gene co-expression and protein interaction networks

**DOI:** 10.1186/1471-2105-11-S3-S7

**Published:** 2010-04-29

**Authors:** Fadi Towfic, Susan VanderPIas, Casey A OIiver, OIiver Couture, Christopher K TuggIe, M Heather West GreenIee, Vasant Honavar

**Affiliations:** 1 Bioinformatics and Computational Biology Graduate Program Iowa State University, Ames, IA, USA; 2 Department of Computer Science, Iowa State University, Ames, IA, USA; 3 Susquehanna University, Selinsgrove, PA, USA; 4 Department of Animal Science, Iowa State University, Ames, IA, USA; 5 Department of Biomedical Sciences, Iowa State University, Ames, IA, USA

## Abstract

**Background:**

Ortholog detection methods present a powerful approach for finding genes that participate in similar biological processes across different organisms, extending our understanding of interactions between genes across different pathways, and understanding the evolution of gene families.

**Results:**

We exploit features derived from the alignment of protein-protein interaction networks and gene-coexpression networks to reconstruct KEGG orthologs for* Drosophila melanogaster, Saccharomyces cerevisiae, Mus musculus* and* Homo sapiens* protein-protein interaction networks extracted from the DIP repository and* Mus musculus* and* Homo sapiens* and* Sus scrofa* gene coexpression networks extracted from NCBI's Gene Expression Omnibus using the decision tree, Naive-Bayes and Support Vector Machine classification algorithms.

**Conclusions:**

The performance of our classifiers in reconstructing KEGG orthologs is compared against a basic reciprocal BLAST hit approach. We provide implementations of the resulting algorithms as part of BiNA, an open source biomolecular network alignment toolkit.

## Introduction

With the advent of fast and relatively inexpensive sequencing technology, it has become possible to access and compare genomes from a wide range of organisms including many eukaryotes as well as bacteria and archea through databases such as GenBank [1], Ensembl [2], PlantGDB [3] and others [4-6]. The availability of genomes from such a wide range of organisms has enabled the comparison and analysis of evolutionary relationships among genes across organisms through the reconstruction of phylogenies [7], common pathways [8,9], and comparing gene functions [10,11]. Of particular interest in this context is the problem of finding genes originating from a single gene from a common ancestor of the compared genomes (orthologs) [12]. Ortholog detection methods present a powerful approach for finding genes that participate in similar biological processes across different organisms, extending our understanding of interactions between genes across different pathways, and understanding the evolution of gene families.

Several sequence-based approaches currently exist for finding orthologous genes among a set of genomes. For instance, one of the simplest methods is to utilize reciprocal best BLAST hits [13] across a set of species to identify orthologs [14]. The COGs (Clusters of Orthologous Groups) approach [15], for example, defines orthologs as sets of proteins that are reciprocal best BLAST hits across a minimum of three species. Another possible approach utilized by databases such as InParanoid [16] and OrthoMCL [17] consists of an iterative BLAST search to construct the reciprocal BLAST hits, and a second step that clusters the reciprocal hits to achieve greater sensitivity. InParanoid uses a pre-defined set of rules to construct its clusters, while OrthoMCL utilizes a sequence-based Markov clustering algorithm for clustering its proteins/genes into ortholog groups. Other approaches, such as PhyOP [18], RAP [19] and others [7,8,10,11] identify orthologous genes/proteins by utilizing phylogenetic analysis to explicitly exploit the evolutionary rates across the species being compared. Such approaches account for the different mutation rates accumulated by the various species being compared, thus allowing greater sensitivity in detecting the pairs of genes/proteins to be classified as orthologous. Methods such as those utilized by Fu et al. consider gene order and rearrangements in detecting orthologs [20]. Recently, with the availability of large-scale analysis of protein-protein interactions, protein-protein interaction networks have also been considered in detecting orthologous genes. Ogata et al. utilized a graph comparison algorithm to compare protein-protein interaction networks and determined orthologs by matching the nodes in the protein-protein interaction graphs [21]. Bandyopadhyay et al. utilized the PathBLAST pathway alignment algorithm to detect orthologs [22]. Another method utilized by databases such as KEGG is to manually construct orthology groups based on a combination of features such as sequence similarity, pathway interactions, and phylogenetic analysis [8,9].

Against this background, we explore a set of graph features that may be utilized in detecting orthologs based on sequence similarity as well as the similarity of their neighborhoods in protein-protein interaction and gene coexpression networks. Furthermore, we construct a set of classifiers that utilize the above features and compare the classifiers to the reciprocal BLAST hits approached for the reconstruction of KEGG orthologs [8]. The basic idea behind our approach is to align a pair of protein-protein interaction/gene coexpression networks and scan the alignment for all possible matches that a node (protein) from one network can pair with in the other network. We then train decision tree [23], Naive-Bayes [24], Support Vector Machine [25], and an ensemble classifier [26] that utilize features from the alignment algorithm to identify KEGG orthologs and we compare the performance of the classifiers to the reciprocal BLAST hit method.

We utilize the alignment algorithms available as part of the BiNA (Biomolecular Network Alignment) toolkit [27] as well as graph features extracted from the aligned networks such as degree distribution, BaryCenter [28], betweenness [29] and HITS (Hubs and Authorities) [30] centrality measures. Our experiments with the fly, yeast, mouse and human protein-protein interaction networks extracted from DIP (Database of Interacting Proteins) [31] as well as the mouse and human gene expression data extracted from NCBFs Gene Expression Omnibus (GEO) [32] demonstrate the feasibility of the proposed approach for detecting KEGG orthologs.

## Materials and methods 

### Dataset

The yeast, fly, mouse and human protein-protein interaction networks were obtained from the Database of Interacting Proteins (DIP) release 1/26/2009 [31]. The sequences for each dataset were obtained from uniprot release 14 [33]. The DIP sequence ids were matched against their uniprot counterparts using a mapping table provided on the DIP website. All proteins from DIP that had obsolete uniprot IDs or were otherwise not available in release 14 of the uniprot database were removed from the dataset. The fly, yeast, mouse and human protein-protein interaction networks consisted of 6, 645, 4, 953, 424 and 1,321 nodes and 20, 010, 17, 590, 384 md 1, 716 edges, respectively. The protein sequences for each dataset were downloaded from uniprot [33]. BLASTp [13] with a cutoff of 1 × 10^-10^ was used to match protein sequences across species. The KEGG (Kyoto Encyclopedia of Genes and Genomes) [8] orthology and uniprot annotations for all species were downloaded from the KEGG website and matched against the uniprot id's for the proteins in the datasets.

For detecting orthologs based on gene-coexpression networks, Affymetrix gene expression data was collected from the GEO database for experiments in selected tissues in pigs (*Sus scrofa*) [34], humans (*Homo sapiens*) [35], and mice (*Mus musculus*) [36]. The collected tissues were: adrenal gland, hypothalamus, spleen, thyroid, liver, small intestine, stomach, fat, lymph node, skeletal muscle, olfactory bulb, ovary, and testes. All expression data were taken from healthy animals. Data from each tissue for a given species were obtained from the same Affy platform. Probe IDs contained in the data were matched with gene IDs, and all available probe expression values for each gene were averaged to obtain one expression value per gene per tissue. Gene sequences were collected from NCBI Entrez [37] and compared across species bidirectionally to identify gene homology. BLASTn [13] with a cutoff of 1 × 10^-10^ was used to match gene sequences across species. The KEGG (Kyoto Encyclopedia of Genes and Genomes) [8] orthology and entrez gene id annotations for all species were downloaded from the KEGG website and matched against the gene id's for the genes in the datasets. The microarray expression measures were utilized to compute the pairwise Spearman rank correlations between all pairs of genes were calculated, with links with with an absolute value correlation cutoff of 0.8 or higher being retained in the resulting weighted graph.

### Graph representation of BLAST orthologs

**Figure 1:**
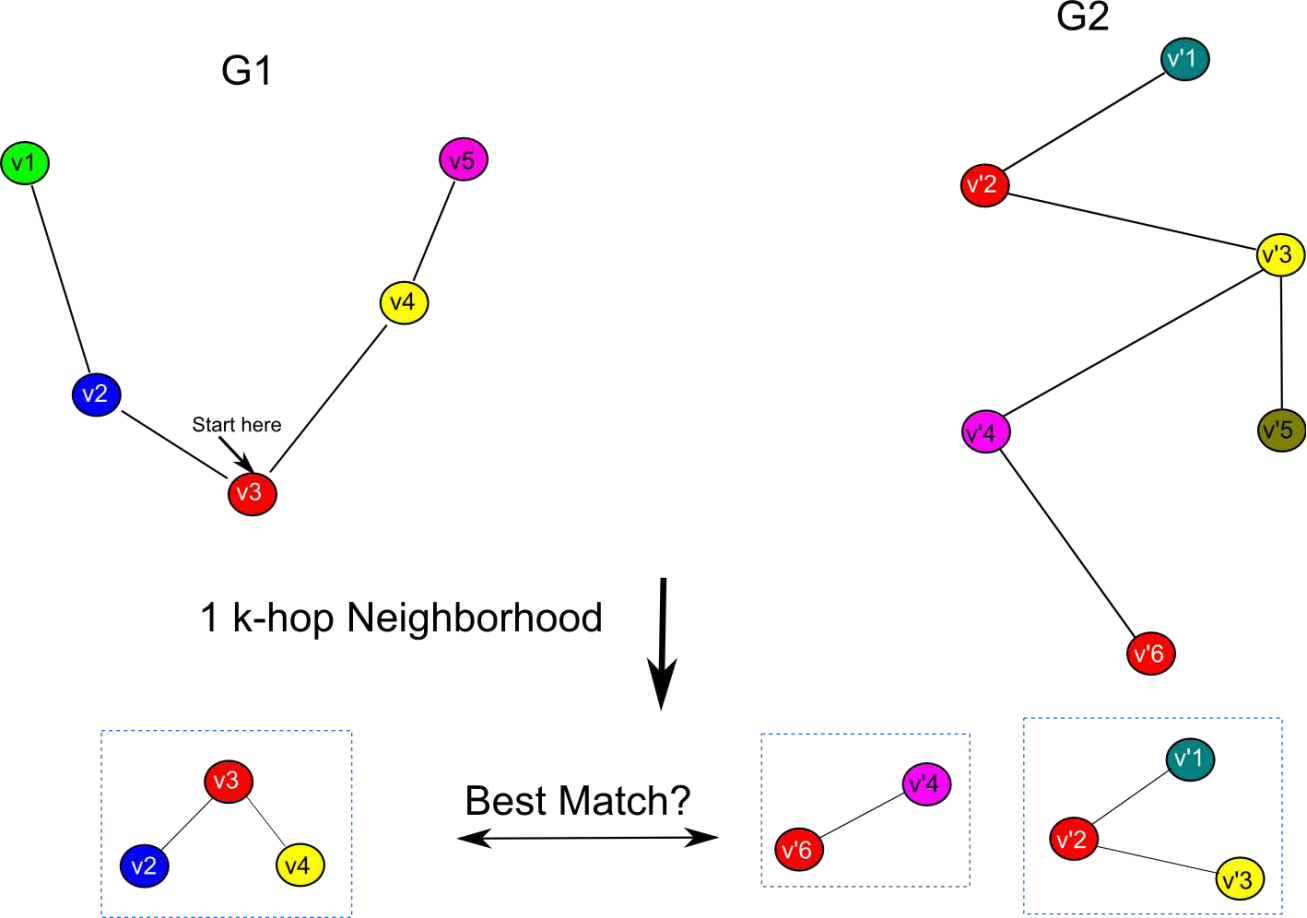
**Graph-based ortholog representation** A schematic of the graph representation of the BLAST orthologs based on protein-protein interaction networks and gene coexpression networks. The networks are represented as two labeled graphs (G1 and G2) with corresponding relationships among their nodes (similarly colored nodes are sequence homologous according to a BLAST search). Nodes from G1 (e.g., v3) are compared to their sequence-homologous counterparts in G2 (e.g., v'2 and v'6) based on the topology of their neighborhood and sequence homology of the neighbors. In the figure, v'2 has the same number of neighbors of v3 and one of the neighbors of v'2 (i.e., v'3) is sequence-homologous to v4. Thus, v'2 is scored higher (more likely to be an ortholog to v3) compared to v'6. Protein-protein interaction networks are represented as unweighted graphs, while gene coexpression networks incorporate weights (as calculated by correlations) into their edges.

The proteins in the DIP protein-protein interaction networks for mouse, human, yeast, and fly as well as the gene coexpression networks for mouse, human and pig from GEO were matched using BLAST as shown in Figure 1. As can be seen from the figure, each protein-protein interaction network or gene coexpression network is represented as a labeled graph (graphs 1 and 2). In the case of protein interaction networks, the graphs (graphs 1 and 2) are unweighted, whereas in the case of gene coexpression networks, the graphs are weighted (where the weights on the edges denote the pairwise correlation in the expression of the corresponding genes). The BLAST similarity scores are taken into account when comparing the neighborhoods around each of the vertices in the graphs to reconstruct the KEGG orthologs. Please note that the sequence homologous nodes across the two graphs in Figure 1 have the same color. A *k*-hop neighborhood-based approach to alignment uses the notion of *k*-hop neighborhood. The *k*-hop neighborhood of a vertex  of the graph *G*_1_(*V*_1_, *E*_1_) is simply a subgraph of *G*_1_ that connects  with the vertices in *V*_1_ that are reachable in *k* hops from  using the edges in *E*_1._ Given two graphs *G*_1_(*V*_1_, *E*_1_) and *G*_2_(*V*_2_, *E*_2_),a mapping matrix **P** that associates each vertex in *V*_1_ with zero or more vertices in *V*_2_ (the matrix **P** can be constructed based on BLAST matches) and a user-specified parameter* k*, we construct for each vertex  its corresponding *k*-hop neighborhood *C_x_* in* G*_1_. We then use the mapping matrix **P** to obtain the set of matches for vertex  among the vertices in *V*_2_; and construct the *k*-hop neighborhood *Z_y_* for each matching vertex  in *G*_2_ and . Let  be the resulting collection of *k*-hop neighborhoods in *G*_2_ associated with the vertex  in *G*_1_. We compare each *k*-hop subgraph *C_x_* in *G*_1_ with each member of the corresponding collection  to identify the *k*-hop subgraph of *G*_2_ that is the best match for *C_x_* (based on a chosen similarity measure). Figure 1 illustrates this process.

### Shortest path graph kernel score

**Figure 2:**
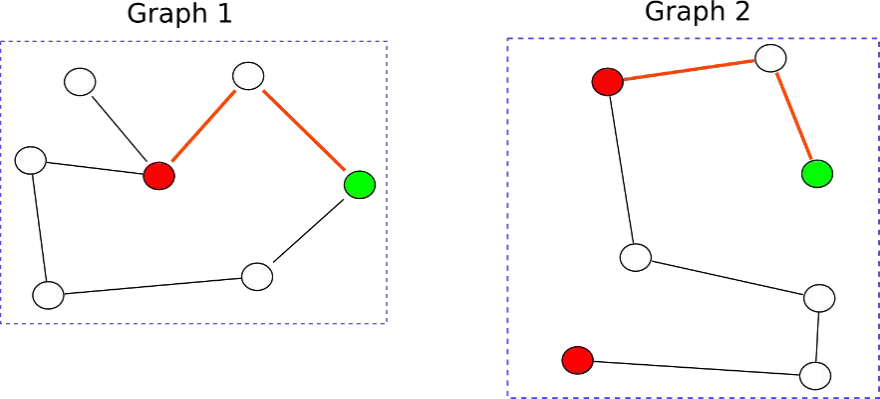
**Shortest path distance graph kernel** An example of the graph matching conducted by the shortest path graph kernel. Similarly colored nodes are sequence homologous according to a BLAST search. As can be seen from the figure, the graph kernel compares the lengths of the shortest paths around homologous vertices across the two graphs (taking into account the weights of the edges, if available). The red edges show the matching shortest path in both graphs as computed by the graph kernel. The shortest path distance graph kernel takes into account the sequence homology score for the matching vertices across the two graphs as well as the distances between the two matched vertices within the graphs.

The shortest path graph kernel was first described by Borgwardt and Kriegel [38]. As the name implies, the kernel compares the length of the shortest paths between any two nodes in a graph based on a pre-computed shortest-path distance. The shortest path distances for each graph may be computed using the Floyd-Warshall algorithm as implemented in the CDK (Chemistry Development Kit) package [39]. We modified the Shortest-Path Graph Kernel to take into account the sequence homology of nodes being compared as computed by BLAST [13]. The shortest path graph kernel for subgraphs and  (e.g., *k*-hop subgraphs, bicomponent clusters extracted from *G*_1_ and *G*_2_ respectively) is given by:

where .  and  are the lengths of the shortest paths between  and  computed by the Floyd-Warshall algorithm. For gene-coexpression network, the Floyd-Warshall algorithm takes into account the weight of the edges (correlations) in the graphs. The runtime of the Floyd-Warshall Algorithm is *O*(*n*^3^). The shortest path graph kernel has a runtime of *O*(*n*^4^) (where *n* is the maximum number of nodes in larger of the two graphs being compared). Please see Figure 2 for a general outline of the comparison technique used by the shortest-path graph kernel.

### Random walk graph kernel score

The random walk graph kernel [40] has been previously utilized by Borgwardt et al. [40] to compare protein-protein interaction networks. The random walk graph kernel for subgraphs  and  (e.g., *k*-hop subgraphs, bicomponent clusters extracted from *G*_1_ and *G*_2_ respectively) is given by:(1)

where **I** is the identity matrix, λ is a user-specified variable controlling the length of the random walks (a value of 0.01 was used for the experiments in this paper),* K_x_* is an *nm* ×* nm* matrix (where *n* is the number of vertices in  and m is the number of vertices in  resulting from the Kronecker product , specifically,(2)

Where ; *p* and *q* are 1 × *nm* and *nm* × 1 vectors used to obtain the sum of all the entries of the inverse expression .

**Figure 3:**
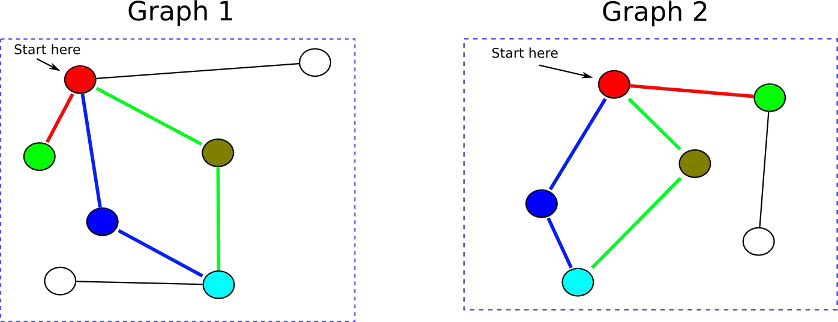
**Random walk graph kernel** An example of the graph matching conducted by the random walk graph kernel. Similarly colored vertices are sequence homologous according to a BLAST search. As can be seen from the figure, the graph kernel compares the neighborhood around the starting vertices in each graph using random walks (taking into account the weights of the edges, if available). Colored edges indicate matching random walks across the two graphs of up to length 2. The random walk graph kernel takes into account the sequence homology of the vertices visited in the random walks across the two graphs as well as the general topology of the neighborhood around the starting vertex.

We adapted the random walk graph kernel to align protein-protein interaction networks by taking advantage of the reciprocal BLAST hits (RBH) among the proteins in the networks from different species [14]. Naive implementation of our modified random-walk graph kernel, like the original random-walk graph kernel [40], has a runtime complexity of *O*(*r*^6^) (where *r* = *max*(*n, m*)). This is due to the fact that the product graph's adjacency matrix is *nm* × *nm*, and the matrix inverse operation takes *O*(*h*^3^) time, where *h* is the number of rows in the matrix being inverted (thus, the total runtime is *O*((*rm*)^3^) or *O*(*r*^6^) where *r* =* max*(*n,m*)). However, runtime complexity of the random walk graph kernel (and hence our modified random walk graph kernel) can be improved to *O*(*r*^3^) by making use of the Sylvester equations as proposed by Borgwardt et al. [40]. Figure 3 illustrates the computation of the random walk graph kernel. The random walk graph kernel can take into account the weight of the edges of the graphs in the case of gene-coexpression networks. The weights for the edges across the two networks must be similar for the two networks to be considered matches.

### BaryCenter score

The BaryCenter score is calculated based on the total shortest path of the node. The shortest path distances for each node in a graph is calculated and the score is assigned to the node based the sum of the lengths of all the shortest paths that pass through the node [28]. More central nodes in a connected component will have smaller overall shortest paths, and 'peripheral' nodes on the network will have larger overall shortest paths.

### Betweenness score

Betweenness is a centrality measure of a vertex within a graph. Vertices that occur on many shortest paths between other vertices have a higher betweenness score than nodes that do not occur on many paths [29].

For a graph *G*_1_(*V*_1_, *E*_1_), the betweenness score for vertex  defined as:

Where  is the number of the shortest paths from  to  and  is the number of shortest paths from  to  that pass through vertex .

### Degree distribution score

The degree distribution score is a simple node importance ranker based on the degree of the node. Nodes with a high number of connections will get a high score while nodes with a smaller number of connections will receive a lower score.

### HITS score

The HITS score represents the "hubs-and-authorities" importance measures for each node in a graph [30]. The score is computed iteratively based on the degree connectivity of the nodes in the graph and the "authoritativeness" of the neighbors around each node. For a graph *G*_1_(*V*_1_, *E*_1_), each node  is assigned two scores:  and . Vertices that are connected to many vertices are marked as hubs, and thus their  scores are large. On the other hand, a vertex that points to highly connected vertices is referred to as an authority and is assigned a high  score. Some nodes can be highly connected (have high  score) and have neighbors that are highly connected (thus, have a high ); such nodes would have a high HITS score.

### Scoring candidate orthologs based on sequence and network similarity

In order to establish orthologs between fly, yeast, human, pig and mouse, the 1 hop and 2 hop shortest path and random walk scores, BLAST score, BaryCenter score, betweenness score, degree distribution score and HITS score were computed for each pair of homologs detected by BLAST (total of 9 features). The BaryCenter, betweenness, degree distribution and HITS scores were combined using Milenkoviæ et al.'s [41] formula for averaging node-based scores in a graph:

Where  and  are the scores for the nodes from *G*_1_(*V*_1_, *E*_1_) and *G*_2_(*V*_2_, *E*_2_), where  and . The above formula produces a normalized score for each node-based feature (BaryCenter, betweenness, degree distribution, and HITS scores) for each pair of homologs while adjusting for any bias in magnitude differences in the scores for the graphs (e.g, *G*_1_ may have much more nodes than *G*_2_, thus the node-based scores for *G*_1_ may be more likely to be greater than the node-based scores for *G*_2_).

### Ortholog detection

We utilized three broad classes of methods for detecting orthologs:

• Reciprocal BLAST hits method [15,16]. The gene/protein sequences for each of the two species (A and B) being compared are BLASTed against each other. This yields for each gene/protein (from species A, the target) a list of candidate orthologs in species B (and vice versa). Suppose the averaged BLAST scores of gene/protein* a_i_* in species A and the genes/proteins* b*_1_, …, *b_m_* in species B are *s*_*i*1_, …, *s*_*im*_. The method predicts the gene/protein in species B that has the highest averaged BLAST score as the ortholog to gene/protein *a_i_* in species A.

• The reciprocal BLAST score-based classifier takes as input the averaged BLAST scores for each possible pair of genes/proteins and outputs a prediction as to whether the pair are orthologous to each other. This method can predict multiple orthologs from species B for each gene/protein from species A (and vice versa).

• The network-based classifier takes as input a vector of pairwise scores (see “Scoring candidate orthologs based on sequence and network similarity" section) computed using the gene-coexpression or protein-protein interaction networks (1 hop and 2 hop Random Walk graph kernel and Shortest Path graph kernel scores as well as the degree distribution, BaryCenter [28], betweenness [29] and HITS (Hubs and Authorities) [30] centrality measures). The classifier outputs a prediction for each pair of genes/proteins as to whether the pair are orthologous to each other. This method can predict multiple orthologs from species B for each gene/protein from species A (and vice versa).

The KEGG [8] ortholog database is used to label the instances in the dataset for training and testing the classifiers.

### Performance evaluation

We compare the performance of the simple methods for detecting orthologs based on reciprocal BLAST hits with the decision tree [23], Naive-Bayes [24], Support Vector Machine [25], and ensemble classifier [26] trained using the BLAST scores as well as the graph-based scores (see "Ortholog detection" section) with 10-fold cross-validation. We used the average ranks of the methods based on their performance estimated using the area under the receiver operating characteristic curve (AUC) to compare their overall performance. Although Demsar's [42] non-parametric test can be used to compare machine learning algorithms, the use of this test requires the number of data sets to be greater than 10 and the number of methods to be greater than 5 [42]. Thus, it cannot be applied directly to our analysis (since we have only 7 datasets and 5 methods). In such a setting, the average ranks of the classifiers provide a reasonable basis for comparing their overall performance [42]. We also report the area under the receiver operating characteristic curve AUC as an additional measure of performance for each of the methods.

## Analysis and results

### Reconstructing KEGG orthologs using BLAST

We compare predictions based only on the BLAST score as well as predictions based on the network features discussed in materials and methods section. The results in Table 1 show the performance of the reciprocal BLAST hits method in reconstructing the orthologs between the fly, yeast, human and mouse datasets from DIP [31]. The last column of of Table 1 shows the performance of the reciprocal BLAST hits method in reconstructing the orthologs between the mouse and human gene-coexpression networks. As can be seen from the table, the reciprocal BLAST method performs fairly well in reconstructing the KEGG orthologs for each dataset. As noted by Bandyopadhyay et al. [22], this may be due to the fact that most ortholog detection schemes, at least in part, depend on sequence homology analysis. For example, although KEGG orthologs use information other than sequence homology (such as metabolic pathway comparison and manual curation) [8], sequence homology plays an important role in the definition of KEGG orthologs. Table 2 shows the performance of classifiers using only the BLASTp scores to detect KEGG orthologs between fly, yeast, mouse and human. The logistic regression classifier in WEKA [23] has the best performance overall (according to the average rank shown in Table 2), however, it does not outperform the reciprocal BLAST hit method shown in Table 1. The results from the gene-coexpression network from mouse and human are comparable overall to the results from the protein-protein interaction networks for the same species.

**Table 1:** BLAST performance for ortholog detection

Datasets	AUC
Mouse-Human (PPI)	90.39
Mouse-Fly (PPI)	92.62
Mouse-Yeast (PPI)	96.14
Human-Fly (PPI)	88.89
Human-Yeast (PPI)	85.63
Yeast-Fly (PPI)	75.03
Mouse-Human (gene-coexpression)	90.40

Performance of the Reciprocal BLAST hit method on the fly, yeast, human and mouse protein-protein interaction datasets from DIP as well as the gene coexpression networks for mouse and human from GEO.

**Table 2:** Classifier performance using BLAST score as the sole feature for ortholog detection

Datasets	Adaboost j48 AUC	NB AUC	SVM AUC	Log. Reg. AUC	Ensemble AUC
Mouse-Human (PPI)	87.79 (4)	90.15 (3)	77.31 (5)	90.29 (2)	90.30 (1)
Mouse-Human (gene-coexpression)	89.80 (4)	70.4 (5)	90.40 (1)	90.40 (1)	90.40 (1)
Mouse-Fly (PPI)	87.58 (4)	88.47 (3)	70.17 (5)	92.01 (1)	88.89 (2)
Mouse-Yeast (PPI)	89.85 (5)	91.89 (2)	90.78 (3)	95.46 (1)	91.45 (4)
Human-Fly (PPI)	81.35 (4)	87.70 (2)	65.90 (5)	88.90 (1)	84.42 (3)
Human-Yeast (PPI)	82.97 (3)	81.26 (4)	63.68 (5)	85.50 (1)	84.19 (2)
Yeast-Fly (PPI)	73.02 (3)	72.49 (4)	56.80 (5)	74.86 (1)	74.48 (2)
*Average Rank* (PPI Only)	3.83	3	4.67	1.17	2.33
*Average Rank* (PPI+GeneCoexpression)	3.86	3.28	4.28	1.28	2.28

Performance of the Reciprocal BLAST hit score as a feature to the decision tree (j48), Naive Bayes (NB) Support Vector Machine (SVM) and Ensemble classifiers on the fly, yeast, human and mouse protein-protein interaction datasets from DIP as well as the gene coexpression networks for mouse and human from GEO. Values in parenthesis are the ranks for the classifiers on the specified dataset.

### Reconstructing KEGG orthologs using sequence, protein-protein interaction network, and gene-coexpression data

**Figure 4:**
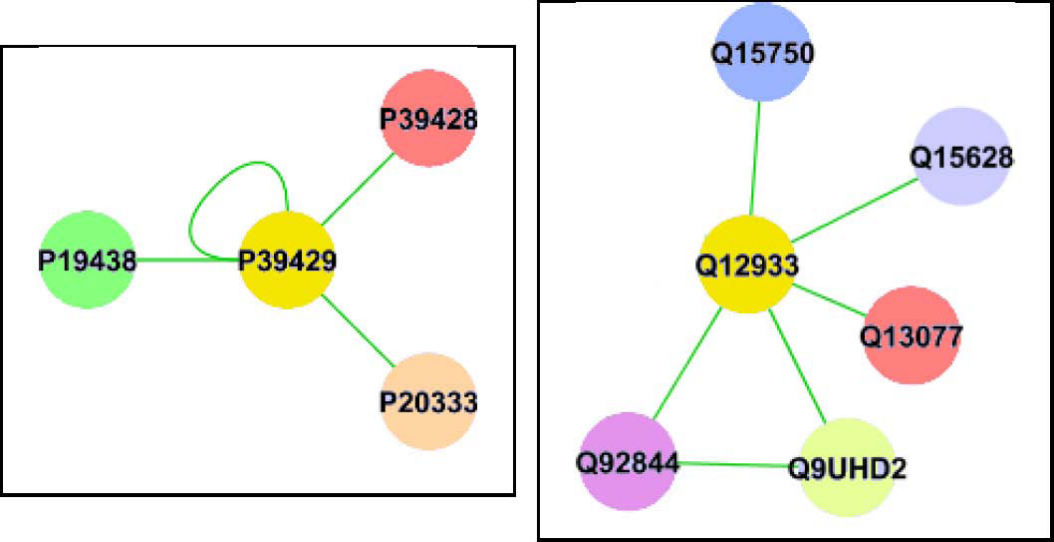
**Example of an ortholog pair detected by the ensemble classifier trained on network features** A sample 1 hop neighborhood around one of the matched orthologs (TNF receptor-associated factor 2 "P39429" in mouse and "Q12933" in human) according to the graph features (**LEFT:** 1 hop network around the "P39429" protein for mouse, **RIGHT:** 1 hop neighborhood around the "Q12933" protein for human). Similarly colored nodes are sequence homologous. The graph properties search for similar topology and sequence homology around the neighborhood of the nodes being compared.

Table 3 shows a comparison of the classifiers trained on the 1 hop and 2 hop Random Walk graph kernel and Shortest Path graph kernel scores as well as the degree distribution, BaryCenter [28], betweenness [29] and HITS (Hubs and Authorities) [30] centrality measures described in materials and methods section. We utilized the approach of Hall et al. [43] as implemented in WEKA [23] to rank the features based on their contribution to the classification performance. We found that the random-walk and shortest-path graph kernel scores were the top two ranked features in terms of their predictive ability. As seen from Table 3, most of the classification methods show some improvement over the classifiers trained only on the BLASTp scores shown in Table 2. Notably, the ensemble classifier on the mouse-human datasets substantially outperforms its BLASTp counterpart on both the protein-protein interaction networks and the gene-coexpression data. Table 4 shows a few representative orthologous pairs that are missed by a regression-based classifier trained on BLASTp scores but are detected by the ensemble classifier trained on the network features and Figure 4 shows the network neighborhood for one of such pairs (the TNF receptor-associated factor 2). This suggests that the combination of sequence homology with network-derived features may present a more reliable approach than simply relying on reciprocal BLASTp hits in identifying orthologs.

**Table 3:** Classifier performance using all features for ortholog detection

Datasets	Adaboost j48 AUC	NB AUC	SVM AUC	Log. Reg. AUC	Ensemble AUC
Mouse-Human (PPI)	95.19 (2)	88.72 (5)	90.78 (3)	89.57 (4)	96.18 (1)
Mouse-Human (gene-coexpression)	89.80 (5)	94.1 (4)	97.50 (1)	97.30 (2)	96.10 (3)
Mouse-Fly (PPI)	90.31 (1)	85.81 (3)	81.28 (4)	80.67 (5)	88.94 (2)
Mouse-Yeast (PPI)	92.04 (3)	85.50 (4)	79.63 (5)	95.60 (1)	95.50 (2)
Human-Fly (PPI)	88.18 (1)	83.10 (4)	75.03 (5)	87.04 (3)	87.20 (2)
Human-Yeast (PPI)	82.83 (2)	81.26 (4)	78.22 (5)	81.57 (3)	84.84 (1)
Yeast-Fly (PPI)	74.52 (1)	69.36 (4)	64.57 (5)	74.33 (2)	72.78 (3)
*Average Rank* (PPI Only)	1.67	4	4.5	3	1.83
*Average Rank* (PPI+GeneCoexpression)	2.14	4	4	2.86	2

Performance of all the combined features (Reciprocal BLAST hit score, 1 and 2 hop shortest path graph kernel score, 1 and 2 hop random walk graph kernel score, BaryCenter, betweenness, degree distribution and HITS) as input to the decision tree (j48), Naive Bayes (NB), Support Vector Machine (SVM) and Ensemble classifiers on the fly, yeast, human and mouse protein-protein interaction datasets from DIP as well as the gene coexpression networks for mouse and human from GEO. Values in parenthesis are the ranks for the classifiers on the specified dataset.

**Table 4:** KEGG ortholog sample tables

Mouse Protein	Human Protein	BLASTp score	RW 1HOP	SP 1HOP	RW 2HOP	SP 2HOP	BaryCenter	betweenness	Degree	HITS
P05627	P05412	481	104	197.35	612	290.27	0.71	0.69	0.01	0.26
P36898	P36894	725	28.13	222.85	90.66	576.51	0.35	0.77	0.01	3.06E- 10
P39429	Q12933	870	48	126.18	150.47	187.45	0.79	0.11	0.01	1.20E- 4

KEGG orthologs detected using the Ensemble classifier utilizing all network features. The orthologs shown in the above table were missed by the BLAST logistic regression classifier.

## Discussion and future work

The availability of genomes from a wide range of organisms has enabled the comparison and analysis of evolutionary relationships among genes across organisms through the reconstruction of phylogenies [7], common pathways [8,9], comparing gene functions [10,11], and network alignment [27,44-52]. Ortholog detection methods present a powerful approach for finding genes that participate in similar biological processes across different organisms, extending our understanding of interactions between genes across different pathways, and understanding the evolution of gene families. We have explored a set of graph-based features that may be utilized for the detection of orthologs among different genomes by combining sequence-based evidence (such as BLAST-based sequence homology) with the network alignment algorithms available as part of the BiNA (Biomolecular Network Alignment) toolkit [27] as well as graph features extracted from the aligned protein-protein interaction networks such as degree distribution, BaryCenter [28], betweenness [29] and HITS (Hubs and Authorities) [30] centrality measures. To the best of our knowledge, this is the first time such an analysis has been carried out based on the comparison of weighted gene-coexpression networks. The features may be used to score orthologous nodes in large biomolecular networks by comparing the neighborhoods around each node and scoring the nodes based on the similarity of their neighborhoods in the corresponding protein-protein interaction and gene-coexpression networks. Classifiers can then be trained using the scores to generate predictions as to whether or not a given pair of nodes are orthologous. Our results suggest that the algorithms that rely on orthology detection methods (e.g., for genome comparison) can potentially benefit from this approach to detecting orthologs (e.g., in the case of the comparison between mouse and human). The proposed method can also help identify proteins that have strong sequence homology but differ with respect to their interacting partners in different species (i.e., proteins whose functions may have diverged after gene-duplication). Our experiments with the fly, yeast, mouse and human protein-protein interaction datasets as well as the gene-coexpression data suggest that the accuracy of identification of orthologs using the proposed method is quite competitive with that of reciprocal BLAST method for detecting orthologs. The improvements obtained using information about interacting partners in the case of the mouse-human data (96.18% for the protein-protein interaction network-based method and 96.10 for the gene-coexpression methods as opposed to 90.31% AUC for the reciprocal BLASTp method) suggest that the proposed technique could be useful in settings that benefit from accurate identification of orthologs (e.g., genome comparison). Using the methods described in this paper, we have predicted the mouse and human orthologs for the pig genes, for which currently there is no KEGG ortholog data (please see Additional file 1 and Additional file 2 for our predictions).

The network neighborhood-based homology detection algorithm is implemented in BiNA (http://www.cs.iastate.edu/~ftowfic), an open source Biomolecular Network Alignment toolkit. The current implementation includes variants of the shortest path and random walk graph kernels for computing orthologs between pairs of subnetworks and the computation of various graph-based features available in the Java Universal Graph Framework library [53] such as the degree distribution, BaryCenter [28], betweenness [29] and HITS (Hubs and Authorities) [30] centrality measures. The modular design of BiNA allows the incorporation of alternative strategies for decomposing networks into subnetworks and alternative similarity measures (e.g., kernel functions) for computing the similarity between nodes. It would be interesting to explore variants of methods similar to those proposed in this paper for improving the accuracy of detection of orthologous genes or proteins using other sources of data (e.g., gene regulatory networks or metabolic networks).

## Competing interests

The authors declare that they have no competing interests.

## Authors contributions

SVP, CO and OC assembled and verified the datasets for the analysis. FT wrote the algorithms, ran the experiments and wrote the initial draft of the manuscript. CT, MHWG and VH supervised the analysis, design of the algorithms and revisions to the manuscript.

## Supplementary Material

Additional file 1 mouse_pig_orthologs.csv

Additional file 2 human_pig_orthologs.csv
